# Evaluation of crystal quality of thin protein crystals based on the dynamical theory of X-ray diffraction

**DOI:** 10.1107/S2052252520007393

**Published:** 2020-06-26

**Authors:** Marina Abe, Ryo Suzuki, Kenichi Kojima, Masaru Tachibana

**Affiliations:** a Graduate School of Nanobioscience, Yokohama City University, 22-2 Seto, Kanazawa-ku, Yokohama 236-0027, Japan; b Precursory Research for Embryonic Science and Technology (PRESTO), Japan Science and Technology Agency (JST), 4-1-8 Honcho, Kawaguchi, Saitama 332-0012, Japan; c Department of Education, Yokohama Soei University, 1 Mihocho, Midori-ku, Yokohama 226-0015, Japan

**Keywords:** protein crystallography, X-ray topography, rocking curves, crystal perfection, oscillatory profiles, dynamical diffraction

## Abstract

Knowledge of X-ray diffraction in macromolecular crystals is important for not only structural analysis of proteins but also diffraction physics. Dynamical diffraction provides evidence of perfect crystals. Until now, clear dynamical diffraction in protein crystals has only been observed in glucose isomerase crystals. We wondered whether there were other protein crystals with high quality that exhibit dynamical diffraction. Here we report the observation of dynamical diffraction in thin ferritin crystals by rocking-curve measurement and imaging techniques such as X-ray topography. It is generally known that in the case of thin crystals it is difficult to distinguish whether dynamical diffraction occurs from only rocking-curve profiles. Therefore, our results clarified that dynamical diffraction occurs in thin protein crystals because fringe contrasts similar to *Pendellösung* fringes were clearly observed in the X-ray topographic images. For macromolecular crystallography, it is hard to obtain large crystals because they are difficult to crystallize. For thin crystals, dynamical diffraction can be demonstrated by analysis of the equal-thickness fringes observed by X-ray topography.

## Abstract 2

The oscillatory profiles of rocking curves and equal-thickness fringes were observed on nearly perfect protein crystals of ferritin, which matches predictions of dynamical diffraction theory.

## 1. Introduction   

X-ray diffraction occurs in every crystalline material. In general, X-ray diffraction phenomena are described by kinematical and dynamical theories (Zachariasen, 1945 ▸; Batterman & Cole, 1964 ▸; Pinsker, 1978 ▸; Authier, 2001 ▸). Kinematical diffraction occurs in small or low-quality crystals. On the other hand, dynamical diffraction only occurs in perfect crystals such as Si, Ge and diamond (Kato & Lang, 1959 ▸; Lefeld-Sosnowska & Malgrange, 1969 ▸; Persson, 1971 ▸; Aldred & Hart, 1973 ▸; Bonse *et al.*, 1977 ▸; Ishikawa, 1988 ▸; Kowalski *et al.*, 1989 ▸; Ishikawa *et al.*, 1991 ▸). In general, almost all crystals are not perfect, *i.e.* some defects exist in crystals. This leads to the conclusion that it is practical to consider only kinematical diffraction for almost all materials.

In order to reveal the protein structures by X-ray diffraction, various methods of growing high-quality protein crystals have been suggested (Chayen *et al.*, 2010 ▸). We examine the relationship between a ‘perfect crystal’ and ‘protein crystals with high quality’. In protein crystallography, crystal quality tends to be commonly evaluated by mosaic width and diffraction resolution. The crystal perfection discussed in this article should be understood to be the physical quality as a simple perfect crystal. The evaluation of several grown crystals has been carried out by X-ray rocking-curve analysis so far (Helliwell, 1988 ▸; Snell *et al.*, 1995 ▸; Boggon *et al.*, 2000 ▸; Volz & Matyi, 2000 ▸; Lübbert *et al.*, 2004 ▸; Koizumi *et al.*, 2013 ▸). Nevertheless, there has been no report on the clear observation of oscillatory profiles of rocking curves by dynamical diffraction. Recently, we revealed that glucose isomerase (GI) crystals can be obtained with high quality (Suzuki *et al.*, 2017 ▸, 2018*a* ▸,*b ▸*). The perfection of these crystals has been characterized by the observation of oscillatory profiles of rocking curves and fringe contrasts, *i.e.* *Pendellösung* fringes, caused by dynamical diffraction of X-rays (Suzuki *et al.*, 2018*a* ▸). The diffraction from this sample leads to the need for dynamical diffraction models; combined kinematical and dynamical diffraction models have also been suggested for a more accurate structural analysis (Suzuki *et al.*, 2018*a* ▸). However, the generality of dynamical diffraction for protein crystals cannot be clarified since the dynamical diffraction has only been observed in GI crystals.

In this study, we demonstrate that the kinematical and dynamical diffraction model applies to very high-quality ferritin crystals. The oscillatory profile of the rocking curves is observed in thin crystals and is in good agreement with that predicted by dynamical diffraction theory. However, the oscillatory profile is also similar to that predicted by kinematical diffraction theory when the crystal thickness is less than the extinction distance. In thin crystals, it is difficult to distinguish whether the oscillatory profile is caused by the dynamical or kinematical diffraction effect. On the other hand, X-ray topographic images clearly show the fringe contrasts similar to *Pendellösung* fringes. The period of the fringes can only be explained by dynamical theory. For evaluation of the crystallinity with dynamical diffraction, especially for thin crystals, it is important to perform not only the rocking-curve measurement but also diffraction imaging over the whole crystals by X-ray topography.

## 2. Methods   

### 2.1. Crystallization of ferritin   

**Figure 1:**
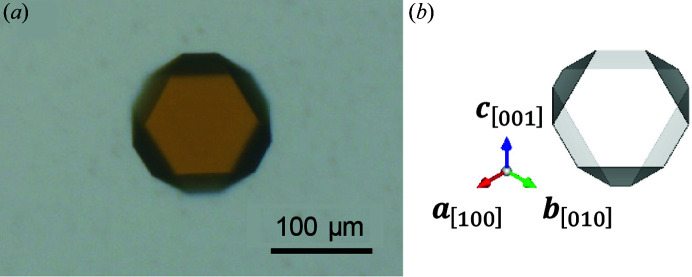
A typical ferritin crystal viewed from the crystallographic direction perpendicular to the (111) plane. (*a*) Optical micrograph and (*b*) corresponding schematic figure prepared with the *VESTA* software (Momma & Izumi, 2011 ▸).

A solution of equine spleen ferritin was purchased from Sigma–Aldrich Co. LLC without further purification. All other chemicals used for preparing solutions were of reagent grade. Ferritin crystals were grown by using macroseeds. First, the seed crystals were obtained using the hanging-drop vapor-diffusion method. The seed crystals were then grown from a solution containing 1.06 mg ml^−1^ ferritin, 0.2 *M* acetic acid–sodium acetate buffer (pH 5.0) and 125 m*M* cadmium sulfate, followed by filtration (pore size 0.1 µm) to remove any impurities and aggregates. The reservoir solution contained 250 m*M* cadmium sulfate. The volumes of the drop and reservoir solution were 3 µl and 1 ml, respectively. After the seed crystals were grown, they were put in a growth droplet (same condition as mentioned above) on siliconized cover glasses (Hampton Research Corp.), where the volume of the droplet was 40 µl. The droplet containing the seed crystals was kept at 20°C for five days so that ferritin crystal was grown on siliconized cover glasses from the seed crystal. Ferritin is a globular protein molecule of 12 nm in diameter and consists of 24 subunits (Vedula *et al.*, 2009 ▸). The molecular weight of ferritin is ∼480 000 Da. The crystal has a face-centered-cubic structure with space group *F*432 and lattice constant *a* = 18.16 nm (Vedula *et al.*, 2009 ▸). Fig. 1 ▸ shows a picture of a typical ferritin crystal as well as a schematic highlighting the typical growth planes.

### 2.2. Measurement of X-ray diffraction   

X-ray topography and rocking-curve measurements were performed at room temperature in the BL14B and BL20B beamlines at the Photon Factory (PF), part of the High Energy Accelerator Research Organization (KEK). All the experiments were carried out in Laue geometry configuration, as shown in Fig. S1 in the Supporting information. Monochromatic beams of λ = 1.2 Å without focusing were selected by adjusting the double-crystal monochromator consisting of a Si (111) crystal at the PF. The incident beam with a size of 3 × 5 mm is enough to measure an entire crystal sample. The grown ferritin crystal on siliconized cover glasses was mounted on a goniometer using wax. The crystal sample on the precision goniometer was rotated about an axis perpendicular to the incident beam with a high-resolution angular step [minimum angular-step width of 0.19 arcsec (5.3 × 10^−5^°)] around the exact Bragg angle of the reflected wave. For the rocking-curve measurement, a high-resolution X-ray CCD camera (Photonic Science X-ray FDI 1.00:1, with an effective pixel size of 6.45 × 6.45 µm) was used as a detector with exposure times of 400 ms, which is enough time to measure the intensity of the diffracted beam. To obtain the high-resolution X-ray topographic images, X-ray films (Agfa D2) were used as detectors with exposure times of 180 s, which is enough time to detect the diffracted images.

## 3. Results and discussion   

**Figure 2:**
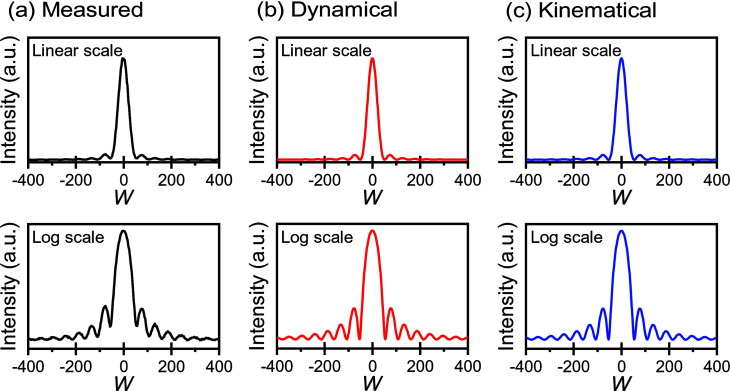
A typical rocking curve for the 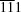 reflection of a ferritin crystal with a thickness of 77 µm, taken with an incident beam with a wavelength of 1.2 Å in BL20B at KEK-PF. (*a*) Measured rocking curves, and theoretical rocking curves for (*b*) dynamical and (*c*) kinematical diffraction shown on linear and logarithmic scales, respectively.

Fig. 2 ▸(*a*) shows a typical rocking curve of ferritin crystals taken with the 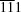 reflection at BL20B at the PF, where the thickness of the ferritin crystal is ∼80 µm. The intensities of the rocking curves are shown on linear and logarithmic scales. The rocking curve is drawn as a function of *W* scale (Authier, 2001 ▸) as opposed to degree or mosaicity, which is more natural for diffraction experiments. The *W* scale is the parameter representing the deviation from the Bragg angle. The *W* scale is given by 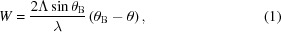
where 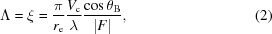
where Λ and ξ are the periods of the *Pendellösung* fringes and the extinction distance in the Laue (transmission) case, respectively. Furthermore, θ_B_ is the Bragg angle, λ is the wavelength of the incident beam, *V*_c_ is the volume of the unit cell, *r*_e_ is the classical electron radius (2.82 × 10^−15^ m) and *F* is the structure factor. From equation (1), the Bragg angle is always at *W* = 0, so it may be easier to compare different reflections. According to equations (1) and (2), *W* is also scaled by the wavelength, the unit-cell volume, and the Bragg angle and structure factor associated with the reflections. *W* for the 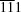 reflection of the ferritin crystal is obtained from equation (1) with θ_*B*_ = 0.328°, λ = 1.2 Å and Λ = 4090 µm, which is calculated from equation (2) using *V*_c_ = 5.99 × 10^−24^ m^3^ and |*F*| = 13 594 (PDB ID 3f32; Vedula *et al.*, 2009 ▸).

It should be noted that an oscillatory profile is clearly observable on the wings of the rocking curve. This oscillatory profile is quite similar to that of GI crystals, as reported previously (Suzuki *et al.*, 2018*a* ▸). The series of X-ray topographic images associated with the oscillatory rocking curve are shown in Movie S1 in the Supporting information.

According to the dynamical theory of X-ray diffraction (Authier, 2001 ▸), the profile of the rocking curve with absorption can be calculated by 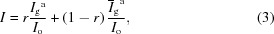
where 
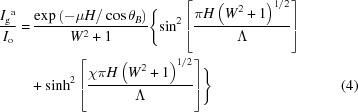
and 
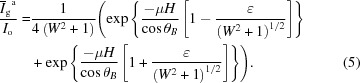
Here, *r* is the ratio of 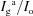 to 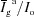 with 0 ≤ *r* ≤ 1, and 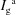, 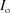 and 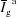 are the diffraction intensity, the incident beam intensity and the averaged diffraction intensity, respectively. The ratio, 1 − *r*, of the average curve corresponds to the degree of smearing or background in the oscillatory curve, which originates from the resolution limit owing to the angular divergence of the beam (Bonse *et al.*, 1977 ▸; Ishikawa, 1988 ▸). Furthermore, μ is the linear absorption coefficient, *H* is the crystal thickness, χ is the electric susceptibility and ∊ is the dielectric constant. Fig. 2 ▸(*b*) shows the calculated profiles of oscillatory rocking curves by dynamical diffraction for the 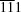 reflection obtained from the ferritin crystal with thickness of 80 µm. The fitting parameters were μ = 2.84 mm^−1^, χ = 0.001 and ∊ = 23 (Authier, 2001 ▸; Suzuki *et al.*, 2018*a* ▸; Zeldin *et al.*, 2013 ▸). The calculated curves are in good agreement with the measured curves.

In contrast, it is well known that even if the crystal perfection is high, kinematical diffraction occurs, when the crystal thickness is quite thin compared with the extinction distance. In the case of protein crystals especially, the volume of the unit cell, *V*_c_, is large and the structure factor, |*F*|, is small compared with those of inorganic materials. Therefore, the extinction distance of protein crystals is several hundreds of micrometres or several millimetres but that of inorganic materials such as Si is around several tens of micrometres. This means that the ratio *H*/Λ, where *H* and Λ are the thickness of the crystals and the extinction distance, respectively, is important to distinguish which predominantly occurs, kinematical or dynamical diffraction.

According to the kinematical theory of X-ray diffraction (Authier, 2001 ▸), the profile of rocking curves can be calculated by 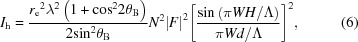
where *I*_h_ is the kinematical diffraction intensity, *N* is the number of unit cells and *d* is the plane distance. Fig. 2 ▸(*c*) shows the theoretical profiles of rocking curves by kinematical diffraction for the 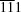 reflection obtained from the ferritin crystal with thickness of 80 µm. This curve is also in good agreement with the measured curves. This means that when the crystal thickness is quite thin compared with the extinction distance, the kinematical and dynamical diffraction cannot be distinguished. In this measurement, the ratio, *H*/Λ, is ∼0.02. In order to clarify the dynamical effect, the dependence of crystal thickness and wavelength is desired (Suzuki *et al.*, 2018*a* ▸). However, it is hard to perform this measurement because of the difficulty of growing large perfect protein crystals.

**Figure 3:**
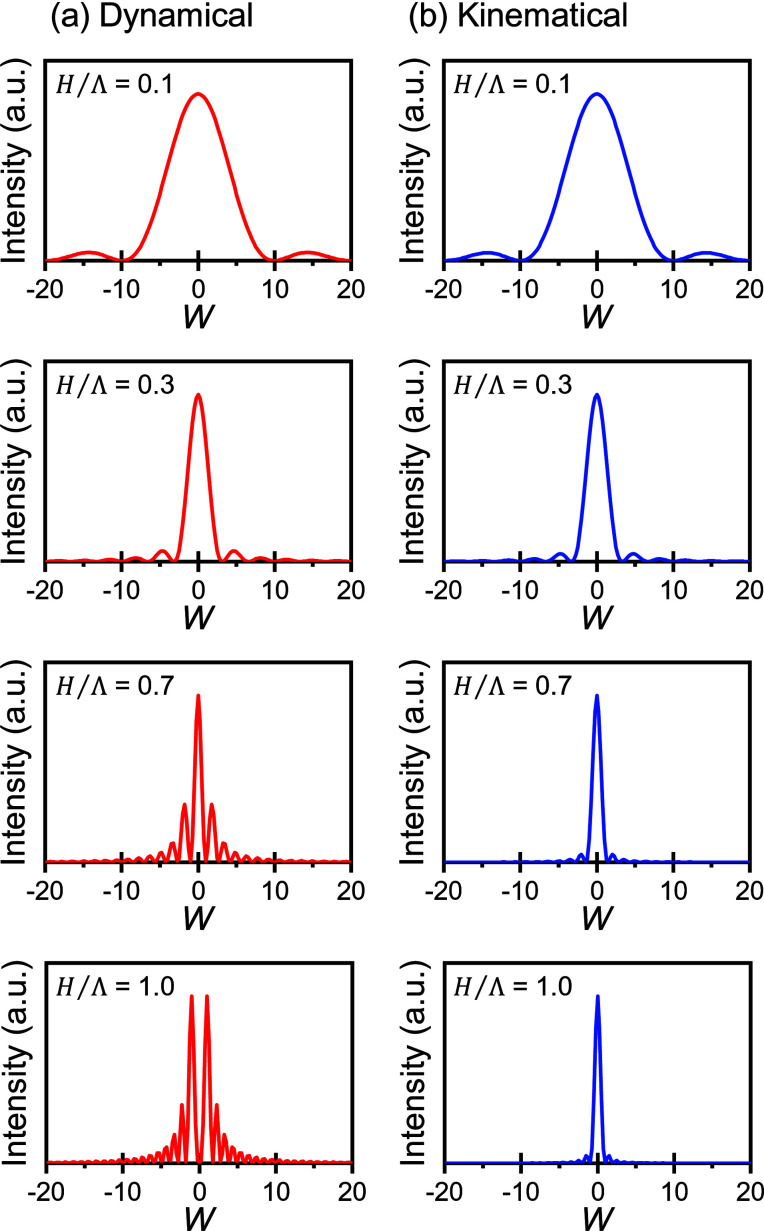
Theoretical profiles of rocking curves for (*a*) dynamical and (*b*) kinematical diffraction. The profile shape depends on the ratio, *H*/Λ, where *H* and Λ are the thickness of the crystals and the extinction distance, respectively.

Fig. 3 ▸ shows the theoretical profiles of rocking curves for dynamical and kinematical diffraction over a range of different values of *H*/Λ. For dynamical diffraction in Laue geometry, the profile shape of the rocking curve depends on the thickness of crystals (Authier, 2001 ▸). The wings of the rocking curves of dynamical diffraction with thicker crystals show higher intensity than that of kinematical diffraction. Additionally, the calculated oscillatory rocking curve exhibits a minimum value at the exact Bragg angle as opposed to the expected maximum value, as shown in Fig. 3 ▸(*a*), when the thickness of the perfect crystal matches to the integer multiple of the extinction distance. Such a oscillatory profile with a minimum value at the exact Bragg angle has been measured for Si single crystals with perfection (Ishikawa *et al.*, 1991 ▸). As shown in Fig. S2, the interesting profile with a local minimum value at the Bragg angle can be also measured for thicker GI crystals (*H*/Λ = 0.91) in which the oscillatory rocking curve caused by dynamical diffraction was first observed in protein crystals (Suzuki *et al.*, 2018*a* ▸). Thus, for thicker crystals, dynamical diffraction is easily demonstrated by the observation of strong oscillation of the rocking curve and/or the local minimum value at the exact Bragg angle. However, it is difficult to judge the dynamical diffraction by only rocking-curve measurement for thinner crystals.

**Figure 4:**
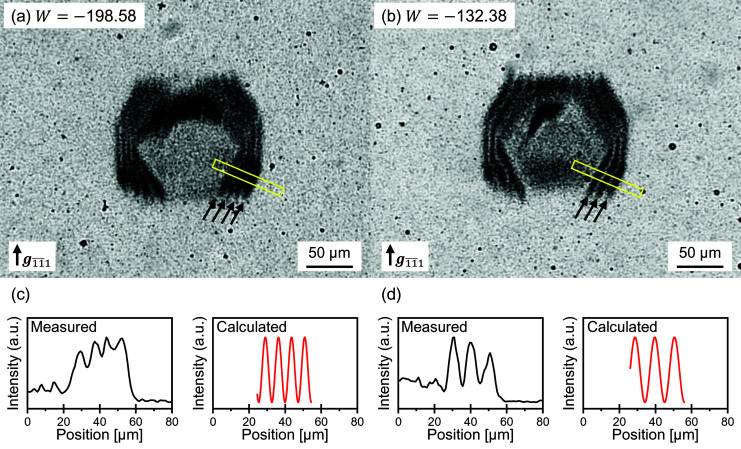
Typical X-ray topographic images of ferritin crystals taken with 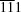 reflection at *W* values of (*a*) −198.58 and (*b*) −132.38, respectively. The numbers of black arrows correspond to those of dark-line contrasts for equal-thickness fringes. The contrast profiles of (*c*) measured and (*d*) calculated intensity of the fringe contrasts at the yellow rectangles in (*a*) and (*b*).

Figs. 4 ▸(*a*) and 4 ▸(*b*) show typical X-ray topographic images of ferritin crystals taken with 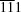 reflection at BL14B at the PF. As seen in Figs. 4 ▸(*a*) and 4 ▸(*b*), the fringe contrasts at the wedge-like edges of the crystals are clearly observed at different values of *W*. The fringe contrasts are caused by the dynamical diffraction of X-rays. Here, the period of fringe contrasts was analyzed. The average periods of the fringes on the wedge-like edges of the crystal shown by the arrows are measured to be 7.78 and 9.95 µm. According to the dynamical theory of X-ray diffraction, the period of the fringes depends on the degree of the inclination of the wedge-like edges at each *W* (Ishikawa, 1988 ▸). Using the angle of the wedge shape as shown in Fig. 1 ▸, the average periods of the peak tops at *W* values of −198.58 and −132.38 are calculated to be 7.28 and 10.9 µm, respectively. The calculated profiles and average values are in good agreement with the measured ones, as shown in Figs. 4 ▸(*c*), 4 ▸(*d*) and Table 1 ▸. This match between measurement and theory is strong evidence of dynamical diffraction. Even though it is difficult to distinguish the origin of the diffraction by measurement of rocking curves with thin crystals, the X-ray topographic image shows clear evidence of the dynamical diffraction. For evaluation of the crystal quality, especially for thin crystals, it is important to perform not only the rocking-curve measurement but also the diffraction imaging with high-resolution X-ray topography. In previous research on Si crystals, the precise structure factor has been experimentally determined from the analysis of equal-thickness fringes observed by using high-collimated X-ray (Kato, 1969 ▸). In protein crystals, it is possible to estimate the precise structure factor by analyzing the fringe contrasts as mentioned above.

**Figure 5:**
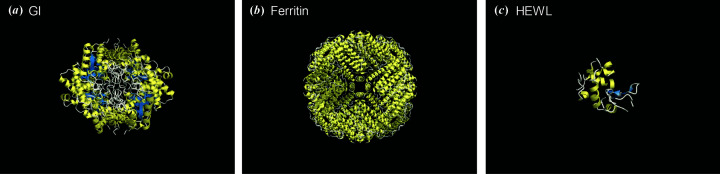
Protein molecules drawn with the *CueMol* software (http://www.cuemol.org/). (*a*) Glucose isomerase (PDB ID 1mnz; Nowak *et al.*, 2002 ▸), (*b*) ferritin (PDB ID 3f32; Vedula *et al.*, 2009 ▸) and (*c*) hen egg-white lysozyme (PDB ID 1lyz; Diamond, 1974 ▸). Note that these are not drawn to scale.

Finally, we consider the origin of the crystal quality of protein crystals. Recently, it has been reported that the crystal quality of hen egg-white lysozyme (HEWL) crystals is improved when the concentration of the precipitant in growth solution is higher (Koizumi *et al.*, 2019 ▸). This is attributed to the faster dynamics of water around the protein molecules (Koizumi *et al.*, 2019 ▸; Aoki *et al.*, 2013 ▸, 2016 ▸). However, the quality might be inferior to that of GI crystals since the oscillatory profiles of rocking curves by dynamical diffraction has not been observed yet. One of the distinct features of GI, ferritin and HEWL molecules is the molecular shape. GI and ferritin molecules have a spherical shape composed of 4 and 24 subunits, respectively, as shown in Figs. 5 ▸(*a*) and 5 ▸(*b*) (Nowak *et al.*, 2002 ▸; Vedula *et al.*, 2009 ▸). On the other hand, HEWL molecule has a croissant shape (not spherical), as shown in Fig. 5 ▸(*c*) (Diamond, 1974 ▸). Therefore, it may be possible to produce the low crystallinity by non-spherical molecular shape. More extensive and quantitative analysis of the relationship between the crystal quality and molecular shapes with other proteins is now in progress.

## 4. Summary   

We have measured the oscillatory profile of rocking curves for thin ferritin crystals. The oscillatory profile can be explained by both dynamical and kinematical diffraction models. For thin crystals, it is difficult to judge from only the rocking-curve profiles whether dynamical diffraction and/or kinematical diffraction occurs. Measurement of the fringe contrasts similar to *Pendellösung* fringes in X-ray topographic images is clearly observed in thin crystals and can be explained by dynamical theory. In brief, dynamical diffraction occurs even in thin ferritin crystals. This knowledge will be useful for the evaluation of crystal quality in thin crystals. Furthermore, the observation of dynamical diffraction using ferritin crystals provides a second example in addition to glucose isomerase. It will be received with considerable interest for a more accurate structural analysis by the physical crystallography community and the macromolecular crystallography community and beyond.

## Supplementary Material

Supporting figures and Supporting movie caption. DOI: 10.1107/S2052252520007393/yu5019sup1.pdf

Movie S1. Serial images of digital X-ray topographs taken as a function of the angle. DOI: 10.1107/S2052252520007393/yu5019sup2.avi

## Figures and Tables

**Table 1:** Measured and calculated average period of the fringe contrasts

		Fringe period (µm)	
*W*	Fringe numbers	Measured	Calculated
−198.58	4	7.78	7.28
−132.38	3	9.95	10.9
