# Supplementary material for: Evaluation of crystal quality of thin protein crystals based on the dynamical theory of X-ray diffraction
Source: IUCrJ. 2020 Jun 26;7(Pt 4):761–6. doi: 10.1107/S2052252520007393 (PMC7340263; doi:10.1107/S2052252520007393)
Supplement: Supplementary file 1 [file m-07-00761-sup1.pdf]

IUCrJ

Volume 7 (2020)

Supporting information for article:

Evaluation of crystal quality of protein crystals with thin thickness based on dynamical theory of X-ray diffraction

Marina Abe, Ryo Suzuki, Kenichi Kojima and Masaru Tachibana

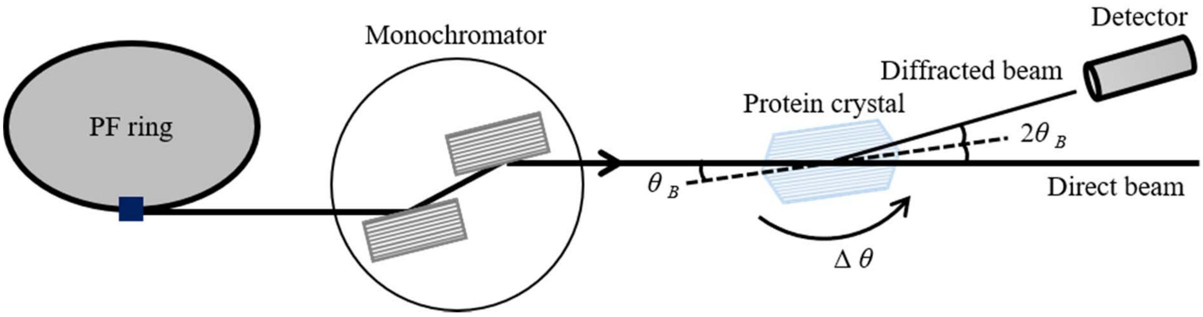

Figure S1 Experimental configuration of X-ray topography. Monochromatic beams of λ = 1.2 Å without focusing were selected by adjusting the double-crystal monochromator consisting of a Si (111) crystal in the PF. The crystal sample was rotated around the exact Bragg angle of reflected wave. High-resolution X-ray CCD camera and X-ray films are used as detector for the rocking curve measurement and X-ray topographic images, respectively.

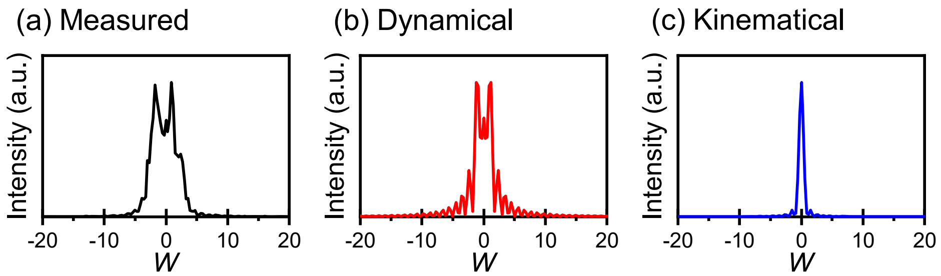

Figure S2 Typical rocking curve for 101 reflection of a GI crystal with a thickness of 540 μm, taken with an incident beam with a wavelength of 1.3 Å. (a) Measured rocking curve, and theoretical rocking curve for (b) dynamical and (c) kinematical diffraction, respectively. Note that the ratio, H/Λ, is 0.91.

Movie S1

Serial images of digital X-ray topographs taken as a function of the angle between $W = \pm 300$ associated with the rocking curve in Figure 2 (a).
